# Cytokines, Hormones and Cellular Regulatory Mechanisms Favoring Successful Reproduction

**DOI:** 10.3389/fimmu.2021.717808

**Published:** 2021-07-28

**Authors:** Marie-Pierre Piccinni, Raj Raghupathy, Shigeru Saito, Julia Szekeres-Bartho

**Affiliations:** ^1^Department of Experimental and Clinical Medicine, University of Florence, Florence, Italy; ^2^Department of Microbiology, Faculty of Medicine, Kuwait University, Kuwait, Kuwait; ^3^Department of Obstetrics and Gynecology, University of Toyama, Toyama, Japan; ^4^Department of Medical Biology, Medical School, Pecs University, Pecs, Hungary; ^5^János Szentágothai Research Centre, Pecs University, Pecs, Hungary; ^6^Endocrine Studies, Centre of Excellence, Pecs University, Pecs, Hungary; ^7^MTA - PTE Human Reproduction Research Group, Pecs, Hungary; ^8^National Laboratory for Human Reproduction, Pecs University, Pecs, Hungary

**Keywords:** progesterone, Th2 dominance, Th 17 cell, cytokines, regulatory T cells

## Abstract

Its semi-allogeneic nature renders the conceptus vulnerable to attack by the maternal immune system. Several protective mechanisms operate during gestation to correct the harmful effects of anti-fetal immunity and to support a healthy pregnancy outcome. Pregnancy is characterized by gross alterations in endocrine functions. Progesterone is indispensable for pregnancy and humans, and it affects immune functions both directly and *via* mediators. The progesterone-induced mediator - PIBF - acts in favor of Th2-type immunity, by increasing Th2 type cytokines production. Except for implantation and parturition, pregnancy is characterized by a Th2-dominant cytokine pattern. Progesterone and the orally-administered progestogen dydrogesterone upregulate the production of Th2-type cytokines and suppress the production of Th1 and Th17 cytokine production *in vitro*. This is particularly relevant to the fact that the Th1-type cytokines TNF-α and IFN-γ and the Th17 cytokine IL-17 have embryotoxic and anti-trophoblast activities. These cytokine-modulating effects and the PIBF-inducing capabilities of dydrogesterone may contribute to the demonstrated beneficial effects of dydrogesterone in recurrent spontaneous miscarriage and threatened miscarriage. IL-17 and IL-22 produced by T helper cells are involved in allograft rejection, and therefore could account for the rejection of paternal HLA-C-expressing trophoblast. Th17 cells (producing IL-17 and IL-22) and Th22 cells (producing IL-22) exhibit plasticity and could produce IL-22 and IL-17 in association with Th2-type cytokines or with Th1-type cytokines. IL-17 and IL-22 producing Th cells are not harmful for the conceptus, if they also produce IL-4. Another important protective mechanism is connected with the expansion and action of regulatory T cells, which play a major role in the induction of tolerance both in pregnant women and in tumour-bearing patients. Clonally-expanded Treg cells increase at the feto-maternal interface and in tumour-infiltrating regions. While in cancer patients, clonally-expanded Treg cells are present in peripheral blood, they are scarce in pregnancy blood, suggesting that fetal antigen-specific tolerance is restricted to the foeto-maternal interface. The significance of Treg cells in maintaining a normal materno-foetal interaction is underlined by the fact that miscarriage is characterized by a decreased number of total effector Treg cells, and the number of clonally-expanded effector Treg cells is markedly reduced in preeclampsia. In this review we present an overview of the above mechanisms, attempt to show how they are connected, how they operate during normal gestation and how their failure might lead to pregnancy pathologies.

## Introduction

The foetus expresses paternal antigens. These are recognized as foreign, but (at least during normal pregnancy) are not attacked by the maternal immune system (1). Immunological recognition of pregnancy initiates a series of changes, which eventually result in a tolerant immunological attitude toward the foetus. Several players of the immune system, such regulatory T cells, NK cells and cytokines contribute to creating a favourable environment for the foetus, and many of these functional alterations are orchestrated and controlled by progesterone.

## Progesterone-Dependent Immunoregulation

In most mammals, progesterone is essential for implantation as well as for the maintenance of gestation. The genomic action of progesterone depends on two nuclear progesterone receptor (PR) isoforms, PRA and PRB (2, 3). Our understanding of their functions stems from studies on progesterone receptor knock-out mice which show that the absence of PRA results in infertility (4, 5), while the PRB isoform mediated effects control mammary gland development (6). PRs are also required for establishing a tolerant immunological milieu in the endometrium (7). Peripheral blood NK cells express both classical PR isoforms (8), and others have also reported on the presence of either nuclear, or G-protein coupled membrane progesterone receptors on lymphocytes (9–12). The latter rapidly alter cell signalling, while nuclear PRs act *via* gene induction. Pregnancy lymphocytes, but not lymphocytes from non-pregnant, women express PRs (13, 14). The majority of PR + cells belonged to the γ/δ T cell population, and treatment of the lymphocytes with anti γ/δ TCR antibody inhibited PIBF- as well as IL-10 production (14).

During normal pregnancy, the percentage of PR-positive cells among circulating lymphocytes increases by gestational age. In peripheral blood of women with recurrent miscarriage, the percentage of PR expressing cells is significantly lower than in that from women with uneventful pregnancies (13, 14), suggesting a relationship between the presence of PR+ lymphocytes and the outcome of pregnancy.

Resting lymphocytes do not express PRs, while lymphocytes exposed to activating stimuli express PRs (15). Lymphocyte immunotherapy for recurrent miscarriage increased the expression of PR on maternal lymphocytes (16) and lymphocytes of transplant patients have also been shown to express PRs (17), Taken together, these data indicate that PR expression in immune cells, is activation-related.

The progesterone-induced blocking factor (PIBF) is one of the progesterone-regulated genes and the resulting protein is accountable for several of the immunomodulatory effects of progesterone. The mRNA transcribed from the PIBF1 gene contains 18 exons, and codes for a 90 kDa protein (18). The 90 kDa form has been shown to have a peri-nuclear localization within the cell, as a component of the peri-centriolar satellite (19, 20). Smaller isoforms produced by alternative splicing are localized in the cytoplasm (18). The full-length molecule and the smaller isoforms convey different functions, the former regulating cell invasion (21, 22), and the latter responsible for the immunomodulatory effects.

Progesterone and PIBF play key roles in establishing the Th2 dominant cytokine balance during normal pregnancy. Progesterone induces naïve T cells to differentiate into Th2-type cells (23), and PIBF signals *via* the IL-4 receptor. Upon engagement, the PIBF receptor forms a heterodimer with the alpha chain of the IL-4 receptor and activates the Jak1/Stat6 pathway (24). Signalling *via* the IL-4 receptor increases Th2 type cytokine production, by which PIBF contributes to the Th2 dominant cytokine pattern during normal pregnancy. PIBF-treated spleen cells of non-pregnant female mice produce significantly more IL-4 and IL-10 than those in the absence of PIBF (25). In lymphocytes from women with recurrent miscarriage progestogens and PIBF induce a Th2 biased cytokine production (26, 27). Furthermore, progestogen treatment of peripheral blood mononuclear cells (PBMC) from women with pre-term delivery induces a Th2 dominant cytokine pattern (26, 27). The T cells of PIBF-deficient pregnant mice differentiate towards Th1 (28).

Several studies suggest that progesterone is an important regulator of Th1/Th2/Th17 and Treg immunity (29–31). Progesterone affects Treg cell generation, either directly or by altering the function of other cells, e.g., by inducing tolerogenic DCs, which leads to the generation of CD4^+^ and CD8^+^ Treg cells (32). Membrane PRs have been detected in Tregs isolated from pregnancy blood, and the number of PR+ Tregs has been shown to increase during gestation and drop before delivery. These data suggest, that the anti-inflammatory action of progesterone through Treg cells might be important for maintaining pregnancy (33)

The relationship between progesterone-dependent immunomodulation and pregnancy outcome has been demonstrated by several animal and clinical studies. PIBF induces decidualization of mouse endometrial stromal cells; furthermore, the peak of PIBF expression in the mouse endometrium corresponds with the implantation window (34).

Depletion of PIBF during the peri-implantation period in mice results in reduced implantation- and increased resorption rates, together with increased decidual and peripheral NK activity; this also results in a significant downregulation of the genes required for T cell activation in CD4+, and an upregulation in CD8+ cells. Simultaneously, in animals treated with anti-PIBF antibodies, the gene for IL-4 is significantly downregulated in CD4+ cells while that of IL-12A is upregulated in CD8+ cells (28). In a preeclampsia rat model, PIBF treatment normalized the Th1/Th2 ratio, reduced the inflammation, corrected the blood pressure and prevented foetal growth restriction (35).

In IVF patients PIBF is detectable in the serum 14 days after embryo transfer (36). During normal human pregnancy, the serum concentrations of PIBF increase with gestational age; lower than normal concentrations predict spontaneous pregnancy termination (37, 38). In women with unexplained miscarriages decidual PR and PIBF expression, as well as serum PIBF concentrations are significantly lower than in healthy pregnant women and all of these parameters show a positive correlation with the number of peripheral γ δ T cells (39).

Taken together, data from both human studies and animal models show that the immunomodulatory action of progesterone is indeed a prerequisite for normal gestation.

## Cytokines and the Maintenance of Pregnancy

Healthy pregnancy is associated with an enhancement of humoral immunity and a downregulation of cell-mediated immunity; this is quite likely due to a down-regulation of Th1 reactivity cytokines and upregulation of Th2 reactivity cytokines (40–42). This shift away from Th1 reactivity and Th1 cytokines is suggested to be conducive to the success of pregnancy, as Th1-type cytokines have a deleterious effect on the conceptus. The administration of single low doses of the inflammatory Th1 cytokines TNFα and IFNγ into pregnant mice causes abortions while the injection of anti-TNFα antibodies reduces abortion rates in an immunologically-driven mouse abortion model (43). TNFα and IFNγ inhibit the outgrowth of human trophoblast cells *in vitro* (44) and induce apoptosis of human trophoblast cells (45).

Spontaneous miscarriage is defined as a pregnancy loss in the first 20 weeks of gestation, while recurrent spontaneous miscarriage (RSM) as two or more miscarriages before the 20th week of gestation (46). About 60% of the cases of RSM are “unexplained”, and researchers have explored immunologic factors that may account for RSM in the absence of genetic, infectious and endocrinologic background. The contribution of maternal humoral and cell-mediated immune factors has been studied in the development of RSM. The foeto-placental unit appears to be invulnerable to attack by humoral immune factors except for anti-phospholipid antibodies which are clearly implicated in a distinct group of RSM cases. Maternal cell-mediated immune effectors that have been studied in maternal peripheral blood and in utero-placental tissues include T lymphocytes, macrophages and natural killer (NK) cells. Cytokines in particular have received a great deal of attention in this context. Considering that cytokines mediate a remarkable range of immune responses including immunity to infections, rejection of allografts, autoimmune diseases and hypersensitivity, it is not surprising that cytokines also affect the maternal-fetal relationship.

When stimulated with human trophoblast antigens, peripheral lymphocytes from women with a history of RSM secrete markedly higher levels of Th1 cytokines with embryotoxic activity (47). Blood lymphocytes stimulated with a mitogen (48) or by co-coculture with placental cells (49) from healthy pregnant women produce significantly higher levels of the anti-inflammatory Th2 cytokines IL-4, IL-5 and IL-10, while women with unexplained RSM produce significantly elevated levels of the pro-inflammatory cytokines IL-2, IFNγ and TNFα. The ratios of inflammatory/anti-inflammatory cytokines are higher in RSM patients, supporting the notion of Th1 or pro-inflammatory cytokine dominance in RSM and a stronger Th2-bias in healthy pregnancy (50). Observations similar to these in the peripheral blood have been made at the maternal-fetal interface; lower levels of T cell clones producing anti-inflammatory cytokines were reported in the decidua of women with unexplained RSM than in the decidua of women with normal pregnancy (51). The expression of pro-inflammatory cytokines is upregulated in the endometrium, while that of anti-inflammatory cytokines is downregulated in women with unexplained recurrent miscarriage as compared to healthy controls (52). It can be inferred therefore that unexplained RSM is associated with a greater bias towards Th1 or pro-inflammatory cytokines (53, 54); and thus, there is ample support for an increased pro-inflammatory cytokine bias in unexplained recurrent miscarriage (47–53).

## Manipulation of Cytokine Profiles

The demonstration of an association between pro-inflammatory cytokines and recurrent miscarriage has spurred research on the downregulation of these cytokines, and upregulation of anti-inflammatory cytokines to create a favourable immunological environment for the foetus. We can consider the use of the pregnancy-related hormone progesterone, which was shown as early as in 1983, to have anti-inflammatory and immunosuppressive properties (55) because of which it was referred to as “Nature’s immunosuppressant” (56). Progesterone suppresses several cell-mediated immune activities including the activation and proliferation of lymphocytes (57) and reverses many of the events that occur during T cell activation (58).

Given that pro-inflammatory cytokines are associated with RSM (47–53) and that progesterone has interesting immunomodulatory effects (55–58), progestogens have been explored for their ability to inhibit or down-regulate the production of Th1/pro-inflammatory cytokines. This includes research on the immunomodulatory capacity of dydrogesterone (6-dehydro-9β, 10α-progesterone) (Duphaston^®^, Abbott Laboratories, USA), an orally-administered progestogen, which is similar to endogenous progesterone in its molecular structure and pharmacological effects, but more potent than natural progesterone, with a high affinity for the progesterone receptor (59).

PBMC from women with a history of unexplained RSM when cultured with dydrogesterone produce significantly lower levels of the Th1 (pro-inflammatory) cytokines IFNγ and TNFα, and significantly higher levels of the Th2 cytokines IL-4 and IL-6 (26). A significant reduction in Th1/Th2 cytokine ratios is observed, indicating a decrease in dominance of Th1 or pro-inflammatory cytokines. The progesterone-receptor antagonist RU486 inhibits the cytokine-modulating effects of dydrogesterone indicating that these effects are mediated *via* the progesterone receptor (26).

A recent study showed that dydrogesterone is able to suppress the production of the pro-inflammatory cytokine IL-17 (60), a powerful chemoattactrant and activator of monocytes and neutrophils. IL-17 treatment of pregnant mice results in foetal loss suggesting that this inflammatory cytokine is antagonistic to pregnancy (61). Elevated levels of IL-17 have been observed in the peripheral blood and decidua of RSM patients (62). The incidence of unexplained RSM is associated with an increase in the level of serum IL-17 and the Th17/Treg ratio cells in peripheral blood and the maternal-fetal interface (63). Thus, dydrogesterone clearly has interesting potent immunomodulatory properties. Dydrogesterone is converted into its major metabolite 20[alpha]-dihydrodydrogesterone (DHD) which has also been shown to be capable of inhibiting the production of IFNγ and TNFα and upregulating the production of IL-10 (64). This is an important beneficial feature for dydrogesterone to be considered as a therapeutic immunomodulator.

The pro-inflammatory cytokines that are downregulated by dydrogesterone are the ones that are deleterious to pregnancy; thus, the ability of progestogens to inhibit the production of these cytokines can be projected to be conducive to healthy pregnancy.

## Supplementation With Dydrogesterone: Clinical Applications

Substantial attention has been focused on exploring the benefits of supplementation with oral progesterone in treating miscarriage (65–67). However, it should be noted that orally-administered progesterone has the disadvantages of being absorbed poorly, having a short biologic half-life (68), losing bioactivity (69) and getting cleared quickly (70). On the contrary, the orally-active progestogen dydrogesterone is a potentially more attractive alternative as it does not suffer from these disadvantages (71). Dydrogesterone continues to retain its immunomodulatory activity even after it is converted to its major metabolite (64). Furthermore, the anti-androgenic properties of dydrogesterone helps to avoid the masculinization of female foetuses (59, 71).

Supplementation with dydrogesterone has been reported to be beneficial in recurrent miscarriage. A randomized, double-blind, placebo-controlled study on dydrogesterone supplementation by Kumar and colleagues demonstrated a significant decrease in the number of miscarriages and an increase in the mean gestational age at delivery (72). Carp conducted a systematic review of randomized trials on dydrogesterone and reported a 10.5% miscarriage rate after dydrogesterone administration and a miscarriage rate of 23.5% in control women; he concluded that there is a significant reduction of 29% in the odds for miscarriage with dydrogesterone when compared to standard care (73). A systematic review and meta-analysis of ten randomized controlled trials by Saccone and colleagues showed that women who received progestogens had a lower risk of recurrent miscarriage (RR 0.72, 95% CI 0.53-0.97) as well as a higher live birth rate (RR 1.07, 95% CI 1.02-1.15) when compared to those who did not (74).

In fact, Schindler suggests that progestogens like dydrogesterone may be considered for preventing or treating a variety of pregnancy complications such as threatened miscarriage, recurrent (habitual) miscarriage, preterm labour and preeclampsia (75).

Thus, dydrogesterone is an immune-modulator that shifts the balance from a Th1 or pro-inflammatory cytokine bias towards a Th2 or anti-inflammatory bias, a milieu favourable to the success of pregnancy. Dydrogesterone may be therefore be considered for effective, safe and orally-administered therapy in unexplained recurrent spontaneous miscarriage.

## IL-17 and IL-22: A Double-Edged Sword for Pregnancy

The Th1/Th2 paradigm has recently been expanded into the Th1/Th2/Th17 and regulatory T (Treg) cells paradigm (42), in which Th2 and T reg cells are responsible for maternal tolerance toward foetal alloantigens, while Th1 and Th17 cells are accountable for spontaneous abortion. However, the role Th17 cells in the Th1/Th2/Th17 and T reg paradigm in pregnancy has not been completely clarified. It appears that the Th17-type cytokines IL-17 and IL-22 together could have both a positive and a negative impact on pregnancy and could thus represent a double-edged sword.

The IL-17 family of cytokines consists of 6 proteins (IL-17A to IL-17F). IL-17A is the hallmark cytokine of Th17 cells, which also produce IL-17F, IL-22 and IL-21. IL-17 is also produced by CD8+cells and by tissue-resident innate cells such as NK, NKT, Tγδ, ILC3 cells (76), by placental macrophages (77), ILC3 (78) and cytotrophoblast and syncytiotrophoblast from normal term pregnancy, spontaneous miscarriage and molar pregnancy (79). IL-1β and IL-23 effectively enhance (80, 81), while IFN-γ, IL-4, and IL-27 suppress (82, 83) the generation of human Th17 cells.

The pathogenic role of IL-17 cells has been suggested in several chronic inflammatory disorders (76, 84). However, its potent inflammatory activity is mostly due to its ability to recruit immune cells, as well as to its synergistic actions with other pro-inflammatory cytokines such as TNF, IL-1β, IFN-γ, GM-CSF, IL-22.

By recruiting and activating neutrophils, IL-17A and IL-17F are significant players in the physiological immune response against extracellular bacteria and fungi (76).

Because of its role in early stage acute allograft rejection (62, 85–87) the possible contribution of IL-17 in foetal allograft rejection, and its accountability for spontaneous abortion has been the focus of intensive research in the last decade. Th17 cells were significantly upregulated, while Treg cells were downregulated in abortion-prone mice. Furthermore, intraperitoneal injection of recombinant IL-17 induced foetal loss in a normal mouse model, and an anti-IL-17 antibody prevented foetal loss in the abortion prone mouse model (61). Thus, IL-17 seems to be a central player of spontaneous abortion in mice. Th17, CD8 T and NKT cells (which have also been identified as the cellular source of IL-17A in pregnant mice), but not γδ T-cells, could have had an impact on fetal development (88).

In the peripheral blood and decidua of patients with unexplained recurrent miscarriage, the number of IL-17-producing CD4+ T cells was found to be increased, whereas that of T reg cells decreased, compared to healthy control subjects (62). IL-27, a suppressor of Th17 cells, decreased in deciduas of patients with unexplained recurrent abortion compared to spontaneous abortion and controls subjects (87). In agreement with these findings, an increase in the Th17/Treg ratio at the maternal-fetal interface in women with unexplained recurrent miscarriage suggested the contribution of Th17 to the loss of maternal-foetal immune tolerance (63, 89).

Although all these findings seem to indicate the deleterious effect of IL-17 on pregnancy, IL-17 accountability for foetal allograft rejection and spontaneous abortion is not evident.

Nakashima et al. (90) reported no significant differences in the number of decidual IL-17+ T helper cells between missed abortion cases without genital bleeding, and normal pregnancies. The increased number of decidual IL-17+ T cells was only observed in abortion complicated with genital bleeding. The authors suggested that after embryonic death, increased IL‐1 or IL‐6 production and decreased TGF‐β production might increase the number of Th17 cells and decrease those of T reg cells in the uterus (42, 90).

None of these prior investigations consider the possibility that Th17 cells might exhibit plasticity. In fact, naive CD4+CD161+ T cells, precursors of Th17 cells (83) could differentiate into Th17, Th17/Th1, and finally into Th1 cells in response to IL-12, or to the prolonged exposure to IL-23 (91, 92). Th17/Th2 cells originate from circulating memory CCR6(+)CD161(+)CD4(+) T cells in the presence of an IL-4-rich microenvironment (93). More recently, a high number of decidual Th17/Th2 cells have been detected at the implantation site in successful pregnancy, whereas Th17/Th1 cells and “proper” Th17 cells were prevalent in RSM during the first trimester miscarriage of normal karyotype foetuses (94). In line with these results, the association of IL-17 and IFNγ production has been demonstrated in the serum of infertile women who had not conceived after embryo transfer in ART (95). Noteworthy is the observation that the levels of IL-4, IL-17A and IL-17F produced by the CD4+ T cells at the implantation site were higher than the levels of these cytokines distant from the embryo implantation site (94). The differentiation of Th17 cells into Th17/Th2 cells at fetal maternal interface is due to soluble HLA-G5, a non polymorphic class I molecule produced by embryo and cytotrophoblast cells (94). Thus, decidual Th17 cells are not necessarily deleterious, and can even be beneficial for pregnancy, if they also produce IL-4.

This raises the question, why prior investigations showed an increased number of IL-17-producing CD4+T cells in the decidua of patients with unexplained RSM compared to healthy control subjects. If Th17, Th17/Th2 and Th17/Th1 cells are not separately investigated within the IL-17+ CD4+ T cell population, the percentage of IL-17-producing CD4+ T cell clones derived from the decidua of RSM patients (59%) is statistically increased compared to the percentage of decidual IL-17-producing CD4+ T cell clones from normal pregnancy (23%) (p=0.000001) (Piccinni MP et al, unpublished data).

**Figure 1:**
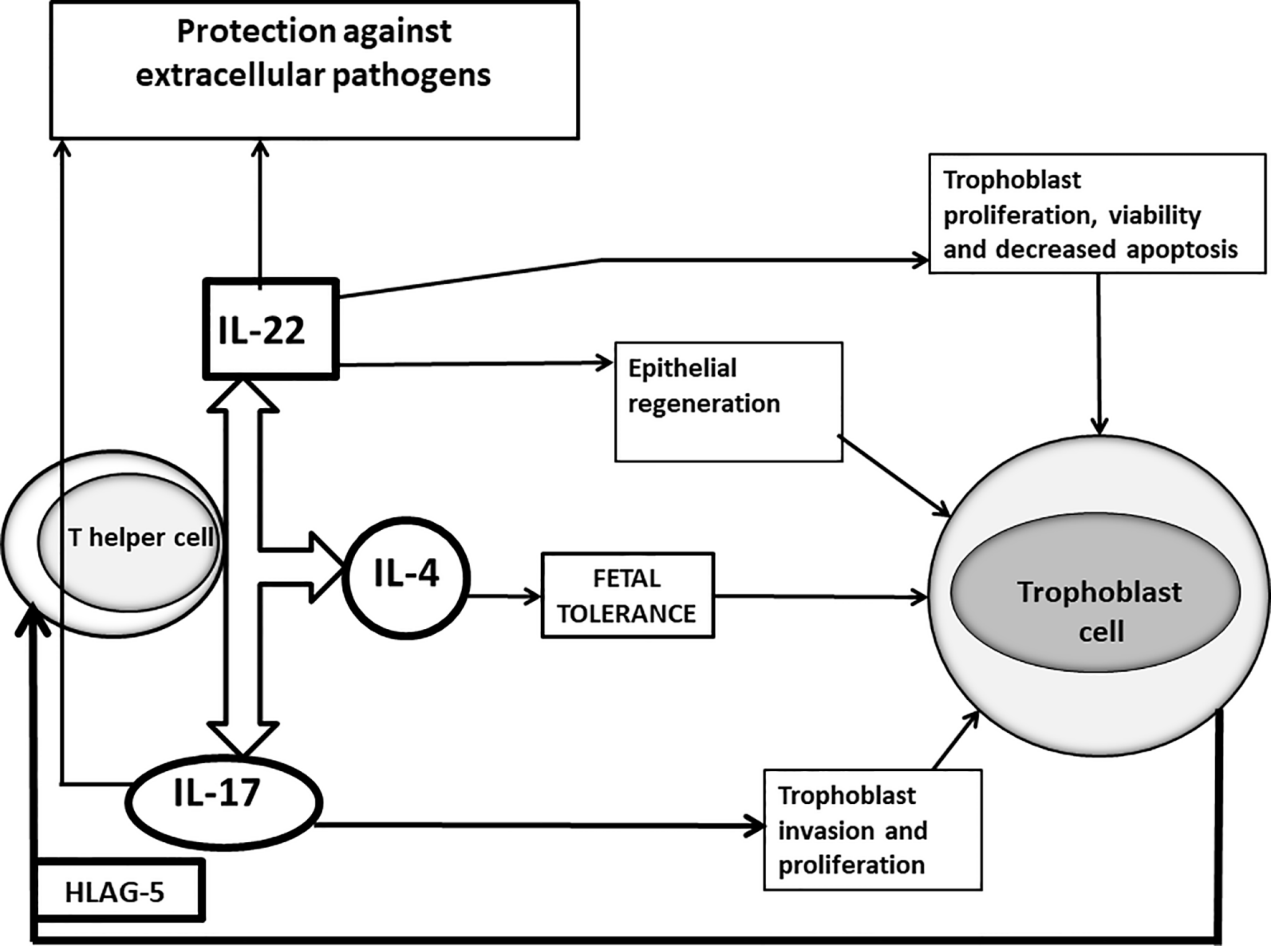
Positive roles of IL-17 and IL-22 at maternal fetal interface of successful pregnancy. In successful pregnancy where IL-17 is produced in association with IL-4 by Th17/Th2 cells stimulated by trophoblast-derived HLA-G5, IL-17 could have a positive influence on pregnancy, by inducing trophoblast proliferation and invasion and by protecting the mother from extracellular pathogens, responsible for miscarriages. IL-22, when it is produced in association with IL-4, could also positively affect pregnancy by repairing damage of the trophoblast cells, by inducing the proliferation, survival and decreased apoptosis of trophoblast cells and by stimulating epithelial cells to secrete antimicrobial peptides supporting the defense against pathogens.

Although these results are statistically significant, these superficial investigations show only a partial image of what happens at fetal-maternal interface and this could lead to incorrect conclusions. In fact, not all the IL-17-producing CD4+ T cells are harmful for pregnancy and IL-17 is not regularly associated with spontaneous abortion. In agreement with this, it has been shown recently that serum IL-17 levels increased in the healthy pregnant women compared to miscarriage (96). The potential role of IL-17 in sustaining pregnancy has already been reported. IL-17 may promote an adequate response to protect the mother from extracellular pathogens (97). Other cells, as γδ T cells producing IL-17 also contribute to the prevention of intrauterine infection (98). More importantly, IL-17 produced by T helper cells favours pregnancy by promoting proliferation and invasion and by inhibiting the apoptosis of human trophoblast cells during the first trimester of pregnancy (99) (**Figure 1**). Thus, the success of pregnancy seems to depend on the increased activity of Th2-Th17/Th2-Treg cells and decreased activity of Th1-Th17/Th1cells.

Interestingly, immunoregulatory factors derived from the placenta could selectively inhibit the simultaneous production of IL-17 and IFNγ by activated T cells (100), and may control the harmful amplification of Th17/Th1 cells in pregnancy.

Based on these results, a more rigorous evaluation of the role of IL-17 in sustaining normal pregnancy is required. More so because emerging data point to a pathogenic role of IL-17 in pre-eclampsia and pre-term birth. The role of IL-17 in preterm labour associated with subclinical infection and increased pro‐inflammatory cytokines in amniotic fluid and decidual tissue remains unclear. In preterm labour, IL-17 produced by T cells has been shown to promote inflammation at the foeto–maternal interface (101). By contrast women with preterm contractions and preterm deliveries showed significantly decreased serum IL-17 levels as compared to normal pregnancies with term deliveries (102). Pre-eclampsia (PE) is associated with exaggerated systemic inflammatory changes and poor angiogenesis. Higher levels of circulating Th17 cells, which could induce strong systemic inflammatory changes and vascular endothelial dysfunction, have been observed in preeclamptic women compared to women with normal pregnancy (42, 103–107). Not only Th17 cells but also Th22 cells have been shown to be involved in the pathophysiology of PE. A positive correlation has been found between the number of Th22 cells and Th17 cells in PE patients (107). Some researchers have reported decreased plasma IL-17 levels (108) or no difference (109) in PE patients compared to healthy pregnant women. Interestingly, others postulated that the increased IL-17 levels observed in patients with preeclampsia are not associated with T cell subpopulations during pregnancy but associated with ILC-3 which, if dysregulated, may pose threats to the foetus (110). The prevalence of IL-17-producing CD4, CD8, ILC-3 and NK cells in pre-eclampsia, might indicate that both the innate and adaptive arms of the immune system could be involved in the development of the exaggerated maternal systemic inflammation observed in this pregnancy-specific disorder (111).

IL-22 is a member of the IL-10 family, which also includes IL-19, IL-20, IL-24, and IL-26 and IFNλ (112, 113). IL-22 is produced by T helper cells (Th1, Th17, Th22), CD8+ T cells and γδ T cells and also by NK cells, ILC3 cells and neutrophils (114, 115). The activation of the aryl hydrocarbon receptor (AHR) is required for the production of IL-22 (116). IL-22 exerts important functions in tissue repair as well as in host defense at mucosal surfaces. However, depending on the target tissue, its effects can be harmful due to its intrinsic pro‐inflammatory activities, which are further enhanced when released together with other pro‐inflammatory cytokines, in particular IL‐17 (117). In murine T helper cells, IL-22 expression is closely related to RORγT and IL-17 expression, and thus IL-22 is considered a Th17 cytokine. However, this relationship is much less evident in the human, where Th1 and Th22 cells are the main sources of IL-22 (117). Furthermore, *in vitro* Th22 cells may develop independently of the Th17 lineage while (similarly to Th17 cells), demonstrating a plasticity toward Th1- and Th2-type cells (118). Under Th1-promoting conditions *in vitro*, *in vivo*, Th22 cells produce IFN-γ, while under Th2 culture conditions *in vitro* they develop into IL-13-producing cells. Consistent with these results, the numbers of skin-homing IL-13– and IL-22–producing Th2/IL-22 and Tc2/IL-22 cells are elevated in subjects with Atopic Dermatitis (known to be driven by strong type 2 immune responses) (119–121).

IL-22 is involved in allograft rejection, by increasing the production of IFNγ by Th1 and Tc1 cells and decreasing the production of IL-10 by Treg and Th2 cells (122). Because of this, IL-22 could play a key role in miscarriage. Findings on the role of IL-22 in RSM are conflicting. Serum levels of IL-22 levels are increased in RSM patients (123, 124), while the expression of IL-22 mRNA is lower in the decidua of RSM patients compared to women with successful pregnancy (125). Human decidual IL-22 is also produced by NK 22 cells and IL-C3, controlled by their interaction with PD-1 ligand expressed by trophoblast cells (126, 127). IL-22 produced by IL-C3 could also be involved in the prevention of preterm labour (128, 129).

The percentage of IL-22-producing CD4+ T cells was higher in the decidua from successful pregnancy, than in the decidua of women with RSM (miscarriage of normal karyotype foetus), suggesting that there is a prevalence of IL-22-producing T helper cells in the decidua of successful pregnancy compared to RSM (130). Four subpopulations of CD4+ T cells producing IL-22 and also producing IL-4 (Th0/IL-22+, Th2/IL-22+, Th17/Th0/IL-22+ and Th17/Th2/IL-22+cells) were associated with successful pregnancy, whereas the only subpopulation of IL-22-producing CD4+ cells associated with RSM (Th17/Th1/IL-22+cells), did not produce IL-4 (130). In addition, serum IL-22 was positively correlated with serum IL-4 in successful pregnancy but not in RSM (130). At the implantation site the percentages of Th17/Th0/+IL-22 and of Th17/Th2/IL-22+CD4+ T cells were higher than those of T cells away from the implantation site (130). Moreover, mRNA expression for IL-4 and IL-22 and their respective transcriptional factors, GATA3 and AHR, were present at the implantation site, whereas the prevalence of mRNA expression for IFNγ and Tbet was observed in patients with ectopic pregnancy (130). Thus, the associated production of IL-22 and IL-4 at the implantation site seems to be essential for the success of pregnancy. The beneficial role of IL-22 for pregnancy has been investigated. As IL-22 is important for epithelial regeneration and wound repair (131, 132), by binding IL-22R1 present on trophoblast cells (133), IL-22 could act at the fetal maternal interface by repairing damage of trophoblast cells. The binding of IL-22 to its receptor could also directly stimulate trophoblast proliferation, survival and decrease its apoptosis (133). IL-22 could also contribute to defence against intrauterine infections responsible for pregnancy loss and for up to 40% of all preterm births, by its ability to induce the secretion of antimicrobial peptides. In fact, supplementation with recombinant IL-22 significantly improved the pregnancy outcome in mice that are challenged with intrauterine lipopolysaccharide treatment (128) (**Figure 1**).

Thus, similar to IL-17, IL-22 could be another cytokine essential for the maintenance of pregnancy when it is produced together with IL-4.

## The Balance Between Immune Activation and Regulation in Pregnancy

Recognition of the semi-allogeneic foetus activates maternal decidual T cells and NK cells (134, 135). Extravillous trophoblasts (EVTs) express polymorphic HLA-C molecules on their surface, and foetal-maternal HLA-C mismatch is associated with decidual T cell activation (136), suggesting that maternal T cells recognize the foetus. Therefore, a mechanism to control rejection is needed for the maintenance of pregnancy. CD4^+^CD25^+^Fox3^+^ regulatory T (Treg) cells play a central role in the establishment and maintenance of allogeneic pregnancy in mice and humans (137, 138). During the implantation period depletion of Tregs results in implantation failure in allogenic-, but not in syngeneic pregnancy, suggesting that Treg cells are essential for successful implantation in mammals (139–141).

Activation of decidual CD8^+^ T cells (135) and paternal antigen-specific CD8^+^ T cells drives fetal resorption in mice (142), suggesting that immunoregulation is necessary to prevent foetal rejection. Kinder et al. reported that PD-1 and LAG-3 on foetal antigen-specific CD8^+^ T cells suppress the cytotoxic activity of CD8^+^ T cells (142). The balance between immunoregulation and immune activation is well-controlled in normal pregnancy, but dysregulated in implantation failure (141), miscarriage with normal karyotype foetuses (42, 62, 143), and preeclampsia (105, 144). Interestingly, decreased effector Treg- and increased effector activating T cell counts are observed in the decidua in miscarriages with normal foetal karyotypes, but not in miscarriages with abnormal fetal karyotypes (143). These findings suggest that immune imbalance might induce RSM of unknown etiology. Similarly, decreased effector Treg cells, increased exhausted Treg cells, and increased activated T cells were observed in preeclampsia (111), indicating immune activation, resulting in maternal and foetal complications.

### Paternal_-_ or Fetal-Antigens Specific Treg Cells and Cytotoxic T Cells in Pregnancy

Paternal antigen-specific tolerance is present during pregnancy and disappears after delivery (145). Treg cells play a central role in the induction of paternal antigen-specific tolerance (146). After delivery, paternal antigen-specific Treg cells decrease in number, but do not disappear, and in the second pregnancy with the same partner, these Treg cells expand more rapidly, than during the first pregnancy (146). A small number of maternal cells cross the placenta and accumulate in foetal lymph nodes (147). Foetal Tregs specific for maternal antigens suppress anti-maternal immunity of the foetus (147). Treg cells present after birth drive postnatal maternal antigen-specific tolerance. In the next generation, microchimeric maternal cells induce maternal antigen-specific tolerance. When a female offspring becomes pregnant with a partner who shares maternal major histocompatibility complex (MHC) antigens, a cross-generational tolerance is established, which prevents foetal loss in mice. Further studies are needed to confirm, whether a similar mechanism exists in humans (148).

Paternal antigen-specific Treg cells accumulate in the uterine draining lymph nodes before implantation, and increase in number in the uterus after implantation (149). Seminal plasma induces the expansion of paternal antigen-specific Treg cells and induces tolerance to paternal alloantigens (149, 150). These findings suggest that the use of condoms and short cohabitation are risk factors for preeclampsia because of insufficient induction of paternal antigen-specific tolerance due to poor seminal priming.

Fetal (paternal) antigen-specific CD8^+^ cytotoxic T cells (CTLs) are detectable in the first trimester and increase in number during pregnancy (54). These CD8^+^ T cells can lyse foetal antigen-expressing cells. The memory type of foetal antigen-specific CD8^+^ T cells persist after delivery (54, 142). However, foetal antigen-specific CD8^+^ T cells are exhausted during secondary pregnancies (151). PD-1 and LAG-3 on CD8^+^ T cells suppress cytotoxicity against foetal cells (142). Thus, the foetus is protected from maternal T cell attack by immune checkpoint molecules and Tregs.

## The Similarity and Difference Between Fetal Antigen- and Tumour Antigen-Specific Treg Cells or Fetal Antigen- and Tumour Antigen-Specific Cytotoxic T Cells

It is challenging to detect foetal (paternal) antigen-specific Treg cells and CTLs in humans. Recent data show that clonally-expanded Treg cells and CTLs are surrogate markers of fetal- or tumour-antigen-recognized Tregs or CTLs (151–161). A single-cell-based T cell receptor (TCR) repertoire analysis method helps to detect clonally-expanded Treg cells or CTLs (151–153). Clonally-expanded Treg cells increase at the foeto-maternal interface (151) and in tumour-infiltrating regions (154, 155, 161). Clonally-expanded Treg cells are scarce in peripheral pregnancy blood, and the similarity of TCR repertoires of effector Treg cells between peripheral blood and decidua is very low (~ 0.2%) (151). However, in cancer the same clonally-expanded Treg cells expressing the same TCR are observed in peripheral blood, and similar TCR repertoires of Tregs are observed between peripheral blood and cancer lesions (161), suggesting that foetal antigen-specific tolerance is localized at the foeto-maternal interface, while tumour antigen-specific tolerance is established in the whole body. This immune condition may lead to distant metastases in cancer. This observation points to differences in immune responses in pregnancy and cancer.

A decreased number of total effector Treg cells is observed in miscarriages with normal foetal karyotype (151), and a decreased number of clonally-expanded effector Treg cells is observed in preeclampsia (151). These findings support the observation that first pregnancy, short cohabitation, and long-term interval from the last delivery are risk factors for preeclampsia but not for recurrent pregnancy loss. These epidemiological risk factors are related to poor induction of paternal antigen-specific Treg cells. Expansion of total Tregs could be considered for use in the therapy of unexplained recurrent pregnancy loss. Alternatively, the proliferation of paternal antigen-specific Treg cells might be used for the treatment of preeclampsia.

In cancer patients with high numbers of clonally expanded intra-tumour Treg cells, the survival rate is low (157), suggesting that clonally expanded Treg cells induce tumour-specific tolerance and tumour cells are thus protected by host T cell attack. This finding shows the similarity between pregnancy and cancer. Anti-PD-1 and anti-CTLA-4 antibodies are helpful for disrupting tumour-specific tolerance, and these antibodies are widely used in cancer treatments.

Clonally-expanded CTLs that recognize fetal antigens or tumour antigens are localized at the feto-maternal interface (152) and tumour tissues (153, 154, 157–160). The proportion of clonally expanded CTLs is higher in the decidua than in the peripheral blood (152). In miscarriage, the total volume of clonally expanded PD-1^-^ CTLs increases in the decidua. The decreased total pool of Treg cells and increased PD-1^-^ clonal CTLs in the decidua could induce fetal rejection, resulting in miscarriage. Alternatively, the number of PD-1^hi^ clonally-expanded decidual CTLs is increased in the third trimester of normal pregnancy, but decreased in preeclampsia (152). Decreased numbers of clonally expanded Treg cells and reduced PD-1 expression on clonally-expanded CTLs could induce fetal rejection, resulting in preeclampsia (**Table 1**).

**Table 1:** Tregs and cytotoxic T cells (CTLs) in normal pregnancy, complicated pregnancy and cancer patient.

	Normal pregnancy		Miscarriage		Preeclampsia	Cancer
	1st trimester	3rd trimester	Abnormal fetal karyotype	Normal fetal karyotype		
**Peripherral blood**						
Total Tregs		↑			↓	↑
effector Tregs		↑			↓	↑
clonal tregs	very few	very few	very few	very few	very few	↑
clonal CTLs	→	→	→	→	→	↑
PD-1*clonal CTLs	→	→	→	→	→	↑
**Decidua or Intratumor**						
Total Tregs	↑	↑↑	↑	↓	↓	↑↑
effector Tregs	↑	↑↑	↑	↓	↓	↑↑
clonal tregs	↑	↑↑	↑	↑	↓	↑↑
Clonal CTLs	↑	↑↑	**?**	↑↑	↑↑	↑↑
PD-1*clonal CTLs	→	↑↑	**?**	→	↓	↑↑

?: unknown, ↑: up-regulated, →: no change, ↓: down-regulated, ↑↑: extremely up-regulated.

In cancer patients, PD-1 is highly expressed in tumour-infiltrating CTLs. The frequency of clonally expanded CTLs is higher in tumour tissue than in peripheral blood, and the clonality rate in tumour-infiltrating PD-1^+^ CTLs is very high, indicating that tumour antigen-specific CTLs express PD-1, resulting in the survival of tumour cells (**Table 1**). This observation shows the similarity between normal pregnancy and cancer, and immunologic milieu in cancer patients with anti-PD-1 therapy is similar to complicated pregnancies such as implantation failure, miscarriage, and preeclampsia. Understanding reproductive immunology and cancer immunology is useful for establishing therapies for miscarriage, preeclampsia and cancer.

## Conclusion

Reproduction is one of the most important factors for the survival of the animal kingdom, and therefore, the system responsible for maintaining gestation is over-insured. The interplay of several parallel mechanisms protects the semi-allogeneic foetus from harmful maternal immune reactions.

Progesterone, is not only indispensable for pregnancy, but also acts as an immuno-steroid. Several studies confirm that progestagen-treatment may have a beneficial effect in recurrent spontaneous miscarriage and threatened miscarriage. The major part of pregnancy is characterized by a Th2-dominant cytokine pattern. Progesterone upregulates the production of Th2-type cytokines and suppresses the production of embryo-toxic Th1 and Th17 cytokines. However, Th17 cells are not entirely harmful for pregnancy, because –depending on the cytokine milieu, they are able to differentiate to either Th17/Th1 or Th17/Th2 cells. Th17/Th2 cells support embryo implantation and pregnancy (94). The Th2 shift induced by progesterone favours the switch of Th17 cells into Th17/Th2 cells, while soluble HLA-G5 produced by embryo and extravillous cytotrophoblast directly induces the differentiation of Th17 cells into Th17/Th2 cells and stimulates the associated production of IL-17 A, IL-17F and IL-4 by T helper cells (94). When produced in association with IL-4, IL-22 can also favour pregnancy and embryo implantation (130) (**Figure 1**).

**Figure 2:**
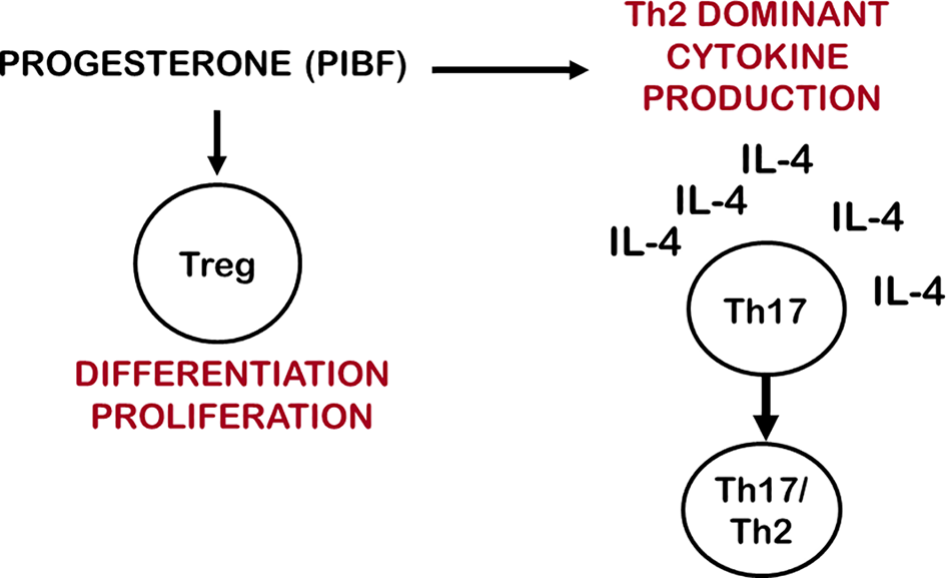
Progesterone affects IL-17 cell differentiation and Treg cell function. By inducing Th2 dominant cytokine production, progesterone creates and IL-4 rich environment, which enables Th17 cells to differentiate to Th17/Th2 cells. *Via* membrane-or nuclear progesterone receptors progesterone stimulates the differentiation and proliferation of clonally expanded Treg cells.

Treg cells express membrane progesterone receptors (mPRα) during pregnancy (162, 163), and human labour may be initiated by a decline in the number of mPR(α+) Treg cells (33). Progesterone has been shown to expand Treg populations by activating nuclear P4 receptors in mice (164) furthermore, it fails to induce Treg cells in nuclear progesterone receptor-deficient T cells (165). These data suggest that both nuclear and membrane progesterone receptors are involved in the mechanisms by which progesterone affects the generation and proliferation of regulatory T cells. Clonally-expanded Treg cells increase at the feto-maternal interface and induce a local, foetal antigen-specific tolerance, while miscarriage and preeclampsia are characterized by a decreased number of clonally-expanded effector Treg cells (**Figure 2**).

## Author Contributions

JS-B wrote about progesterone-dependent immunoregulation. RR summarized the role of cytokines in pregnancy and the use of progestogens modulating cytokine profiles. M-PP reviewed the role of IL-17 and IL-22 in pregnancy. SS compared the role of regulatory T cells in pregnancy and tumours. All authors contributed to the article and approved the submitted version.

## Funding

This work was supported by EFOP-3.6.3.-VEKOP-16-2017-00009, PTE ÁOK-KA 2017–22 EFOP-3.6.1.-16-2016-00004, Thematic Excellence Program 2020 - Institutional Excellence Sub-programme of the Ministry for Innovation and Technology in Hungary, within the framework of the second thematic programme of the University of Pecs to JS-B, AMED P18gk0110018h0003 and JP20gk0110047h0002. to SS.

## Conflict of Interest

The authors declare that the research was conducted in the absence of any commercial or financial relationships that could be construed as a potential conflict of interest.

## Publisher’s Note

All claims expressed in this article are solely those of the authors and do not necessarily represent those of their affiliated organizations, or those of the publisher, the editors and the reviewers. Any product that may be evaluated in this article, or claim that may be made by its manufacturer, is not guaranteed or endorsed by the publisher.
